# Satisfaction with high-resolution anoscopy for anal cancer screening among men who have sex with men: a cross-sectional survey in Abuja, Nigeria

**DOI:** 10.1186/s12885-020-6567-3

**Published:** 2020-02-05

**Authors:** Rebecca G. Nowak, Chinedu H. Nnaji, Wuese Dauda, Andrew Mitchell, Oluwole Olaomi, Paul Jibrin, Trevor A. Crowell, Stefan D. Baral, Nicaise Ndembi, Manhattan E. Charurat, Joel M. Palefsky, Søren M. Bentzen, Kevin J. Cullen, on behalf of the TRUST/RV368 Study Group, Manhattan Charurat, Julie Ake, Sylvia Adebajo, Stefan Baral, Erik Billings, Trevor Crowell, George Eluwa, Charlotte Gaydos, Sosthenes Ketende, Afoke Kokogho, Hongjie Liu, Jennifer Malia, Olumide Makanjuola, Nelson Michael, Nicaise Ndembi, Jean Njab, Rebecca Nowak, Oluwasolape Olawore, Zahra Parker, Sheila Peel, Habib Ramadhani, Merlin Robb, Cristina Rodriguez-Hart, Eric Sanders-Buell, Sodsai Tovanabutra, Erik Volz

**Affiliations:** 1 Division of Epidemiology and Prevention, Institute of Human Virology, University of Maryland School of Medicine, 725 W. Lombard Street, Baltimore, MD 21201 USA; 2 Department of Epidemiology and Public Health, University of Maryland School of Medicine, Baltimore, MD USA; 3 University of Maryland Greenebaum Comprehensive Cancer Center, University of Maryland School of Medicine, Baltimore, MD USA; 4 Institute of Human Virology Nigeria, Abuja, Nigeria; 5 National Hospital, Abuja, Nigeria; 6 U.S. Military HIV Research Program, Walter Reed Army Institute of Research, Silver Spring, MD USA; 7 Henry M. Jackson Foundation for the Advancement of Military Medicine, Bethesda, MD USA; 8 Johns Hopkins School of Public Health, Baltimore, MD USA; 9 Department of Medicine, University of California, San Francisco, CA USA

**Keywords:** High-resolution anoscopy, Acceptability, MSM, Anal cancer screening, LMIC

## Abstract

**Background:**

Men who have sex with men (MSM) living with HIV are at increased risk for anal cancer. We evaluated satisfaction with first-time anal cancer screening using high resolution anoscopy (HRA) as a cross sectional survey among men who have sex with men (MSM) attending a community-engaged clinic in Abuja, Nigeria.

**Methods:**

Between March and August 2017, 342 MSM underwent screening and 307 (89%) completed a satisfaction survey that evaluated 8 domains related to expectations, convenience, staff interpersonal skills, physical surroundings, technical competence, pain/discomfort, general satisfaction, and intention to re-screen if symptomatic. The 22-item questionnaire used 5-point Likert scales ranging from 1 (strongly disagree) to 5 (strongly agree). For each domain, responses to specific items were averaged, aggregated, and converted to a 100-point scaled score (SS) with 25 and 75 corresponding to disagree and agree, respectively.

**Results:**

Median age was 24 years (interquartile range [IQR]: 22–28), median years since anal coital debut was 7 (IQR: 4–12), and 58% (95% confidence interval [CI]: 52–64%) were living with HIV. Despite respondents reporting pre-procedure anxiety (SS:73), most were comfortable with the setting and procedure and reported overall satisfaction (SS:74–76). Willingness to undergo future screening had the lowest score (SS:69) within the general satisfaction domain. The lowest scoring domains were pain/discomfort (SS:57) and agreement to re-screen if symptomatic (SS:59), which correlated with lower overall satisfaction (*p* < 0.001). Domain responses did not differ by HIV infection after adjusting for multiple comparisons (*p* > 0.006) or number of anal biopsies (all *p* > 0.05).

**Conclusions:**

Overall, HRA was satisfactory for those naïve to screening but moving forward necessitates monitoring levels of discomfort with pain scales and normalizing dialogue around clinical symptoms of anal cancer and overall anal health to sustain future screening.

## Background

Persistent high-risk human papillomavirus (HPV) infection is associated with the development of high-grade squamous intraepithelial lesions (HSIL), particularly among men who have sex with men (MSM) living with HIV. HSIL has the potential to regress with an effective immune response, but the 5-year progression rate of anal cancer from HSIL has been estimated to be 2–3% among men living with HIV [1, 2]. A recent study suggested anal cancer incidence is rising steeply for younger black men beginning at age 30 and is 5-fold higher among those born in the mid-eighties compared to those born in mid-forties in the United States [3]. Without clinical evidence demonstrating that screening prevents anal cancer there are no national guidelines to standardize screening practices and set age limits for screening [4]. However, recommendations exist for those at highest risk, including MSM and persons living with HIV [5–8]. High resolution anoscopy (HRA) and directed biopsy are currently used for early detection of HSIL, which if present, can be treated or actively monitored for natural regression. These strategies potentially improve long-term health outcomes given the complications of treating invasive cancer [9, 10].

During HRA examinations, a physician observes a magnified and illuminated view of the anal canal with a colposcope. A lubricated tube, known as an anoscope, is manipulated around the circumference to flatten anal folds for identification and treatment of HSIL. The procedures may be perceived as invasive and cause pain, resulting in an unfavorable response to screening, ultimately reducing participation and sustainability of a screening program. A few studies have evaluated patient level satisfaction with HRA and found that participants who underwent the procedure generally reported little pain and 92–99% agreed to future HRA examinations [11–14]. However, the prior satisfaction surveys were conducted in developed countries such as the United States, United Kingdom, and Canada, where the majority of screening programs exist for at-risk populations [12, 15, 16]. In sub-Saharan Africa, where there is an unknown burden of anal cancer [17, 18], the limited knowledge about the disease and lack of screening programs could impact acceptability. This is particularly relevant in an environment where engagement of MSM in healthcare settings is challenged by pervasive stigma against same-sex practices [19, 20]. This study assessed satisfaction with anal cancer screening using HRA among MSM living with or at risk for HIV in Abuja, Nigeria.

## Methods

### Study population and screening

Anal cancer screening was conducted as a primary analysis for a year in the TRUST/RV368 cohort in Abuja, Nigeria [21–23]. The purpose of the TRUST/RV368 cohort, was to engage men who have sex with men in an HIV treatment-as-prevention study at “trusted” community centers that specialized in the clinical care and sexual health needs of sexual and gender minorities. In brief, TRUST/RV368 participants were educated on the rationale and procedures of the anal cancer screening study. Men who volunteered to participate in the screening study, aged ≥18 years, and provided informed consent in English or Hausa were enrolled. Exclusion criteria included any medical condition that would increase risk associated with HRA or anal biopsy, such as a bleeding disorder or an allergy to lidocaine or iodine. Enrolled men underwent swabbing for cytology and future HPV testing, digital ano-rectal examination (DARE), HRA and HRA-directed biopsies of gross abnormalities for histologic evaluation. Two percent lidocaine was mixed with lubricant and used for DARE and HRA. Five percent acetic acid and Lugol’s iodine were used to visualize abnormalities and only men with acetowhite lesions were biopsied. MSM diagnosed with HSIL were offered ablative treatment with an infrared hyfrecator. Beginning in March 2017 through August 2017, the self-administered satisfaction questionnaire was available for completion after HRA screening. Additional demographic, clinical and behavioral data were obtained from interviewer-administered questionnaires collected by the TRUST/RV368 cohort and the anal cancer screening study.

### Screening assessment

The 22-item questionnaire evaluated 8 domains relating to satisfaction with anal cancer screening: expectations, convenience, staff interpersonal skills, physical surroundings, perceived technical competence, pain/discomfort, general satisfaction, and intention to screen if symptomatic. Eighteen of the questions were previously used by Kwong et al. to assess patient satisfaction during the implementation of an anal cancer screening program at the University of Colorado [11]. They modified a validated scale for assessing satisfaction with colon cancer screening [24] to reflect anal cancer screening procedures. They reviewed the scale’s internal consistency with factor analysis and Cronbach’s alpha and found high factor loading for two subscales, the satisfaction with clinical program and satisfaction with exam experience (Cronbach α = 0.866 and α = 0.873, respectively) with values of at least 0.6 indicative of internal consistency. The third subscale was distress and included only two questions (anxious and embarrassed by the procedure) and had lower internal consistency (Cronbach α = 0.550) [11]. In our study, an additional 4 questionnaire items were added. One item, “Observing the procedure on the monitor made me more comfortable during the exam” was added to evaluate whether being engaged visually relaxed patients during the exam. Three items were added to assess likelihood of pursuing screening if presented with clinical symptoms of anal cancer (“If I had rectal or abdominal pain; bleeding either from wiping after a bowel movement or on my stool; constipation or straining or smaller/unusually shaped stools, then I would seek screening”). All questionnaire items were posed on a 5-point Likert scale ranging from 1 (strongly disagree) to 5 (strongly agree). Reverse coding was applied to some of the responses to maintain a consistent direction of response favorability for the analyses. These included the following statements: “I found it hard to find a convenient time to come for screening”, “The staff seemed to hurry me through too quickly”, “The staff used words that were hard to understand”, “The physician was too rough when performing the screening”, “I had a lot of pain during the procedure”, “The procedure caused me great discomfort” and “I was embarrassed by the procedure”.

### Statistical analyses

Distributions of baseline demographics by HIV status were evaluated using Pearson’s Chi-square and Fisher’s exact tests. Data on age, years since anal coital sexual debut, sexual position (insertive only, receptive only or both) any transactional sex in the past year, and laboratory diagnosed HIV and/or rectal sexually transmitted infections were obtained from TRUST/RV368 cohort [21, 22]. Transactional sex was defined as having exchanged anal or oral sex for things wanted or needed such as money, drugs, food, shelter or transportation [25]. HIV was diagnosed from finger stick blood samples using a parallel testing algorithm [26]. Nucleic acid amplification diagnoses of rectal *Neisseria gonorrhoeae* and *Chlamydia trachomatis* [26] were further categorized as none, individual infections, or both. Data on smoking, lifetime number of receptive partners, external warts, number of anal biopsies and anal biopsy results were obtained from the anal cancer screening study [23]. Anal biopsy results were a composite variable defined as worst diagnosis from either cytology or histology. A sensitivity analysis was conducted to look at the distribution of demographic, behavioral and clinical characteristics for those who responded to the satisfaction survey as compared to the non-responders.

Means and standard deviations of each 5-point Likert response and for the overall eight evaluated domains were calculated and converted to an aggregated scaled score (0, 25, 50, 75, 100) using the following formulas: scaled mean = [raw mean-1] × 25 and scaled standard deviation = raw standard deviation* [scaled mean/raw mean]. To assess concordance between responses within domains, the coefficient of variation was calculated by dividing the standard deviation of the raw mean scores by the overall mean and multiplying by 100. To evaluate differences in satisfaction by HIV status or recent HIV diagnosis, domain means were compared using Wilcoxon rank-sum test and adjusted for multiple comparisons using Bonferroni’s correction. To evaluate differences in satisfaction by number of biopsies experienced during HRA, the domain means were dichotomized as having any agreement (raw mean ≥ 3.5) or no agreement (raw mean < 3.5). Proportional differences in agreement by number of biopsies were evaluated using Chi-square and Fisher’s exact tests. We also explored whether the two lower scored domains (pain/discomfort and intention to screen if presented with clinical symptoms of anal cancer) correlated with overall satisfaction and with each other using Spearman’s rank correlation. Data were analyzed using Stata Statistical Software: Release 13 (Stata Corp LP, College Station, TX) and will be made available upon request.

## Results

Of 355 MSM offered HRA, 13 (3%) refused, 342 (96%) engaged, and 307 (86%) completed the self-administered satisfaction survey after HRA and were included in these analyses. The majority of men who completed the satisfaction survey were under the age of 35 (90%) and 58% were living with HIV (95% confidence interval [CI]: 53–64%). Median years since anal coital debut was 7 (interquartile range [IQR]: 4–12). Of the 178 living with HIV, 60% self-reported being told by a doctor that they had HIV prior to entry into TRUST/RV368 and 40% were newly diagnosed at enrollment in TRUST/RV368. Twenty-two percent of participants were current smokers and an additional 5% had smoked in the past. MSM living with HIV had been engaged in anal sex for longer and with more lifetime partners as compared to MSM not living with HIV (Table 1). MSM living with HIV were more likely to have external warts and anal dysplasia as compared to those not living with HIV (Table 1). Compared to men living with HIV, MSM without HIV were younger and were more likely to report exclusively insertive sexual positioning. In the sensitivity analysis, responders to the satisfaction survey were similar to non-responders on all demographic, clinical and behavioral characteristics listed in Table 1 (all *p* > 0.05, data not shown).

**Table 1:** Characteristics of Men who Have Sex with Men Living with HIV or at Risk for HIV Undergoing High-Resolution Anoscopy

	Total *N* = 307 *N* (%)	HIV+ *N* = 178 *N* (%)	HIV- *N* = 129 *N* (%)	P*
Age (years)				**< 0.01**
≤ 24	136 (44.3)	63 (35.4)	73 (56.6)	
25–34	139 (45.3)	96 (53.9)	43 (33.3)	
≥ 35	32 (10.4)	19 (10.7)	13 (10.1)	
Ever married				0.73
No	272 (89.8)	158 (89.3)	114 (90.5)	
Yes	31 (10.2)	19 (10.7)	12 (9.5)	
Ever smoked				0.68
No	223 (73.1)	131 (74.0)	92 (71.9)	
Yes	82 (26.9)	46 (26.0)	36 (28.1)	
Currently smoke				0.70
No	240 (78.4)	141 (79.2)	99 (77.3)	
Yes	66 (21.6)	37 (20.8)	29 (22.7)	
Years since anal sexual debut				**< 0.01**
≤ 7	150 (51.4)	70 (41.4)	80 (65.0)	
≥ 8	142 (48.6)	99 (58.6)	43 (35.0)	
Lifetime No. of receptive partners				**< 0.01**
≤ 10	217 (72.3)	108 (62.1)	109 (86.5)	
≥ 11	83 (27.7)	66 (37.9)	17 (13.5)	
Sexual position				**< 0.01**
Insertive	64 (21.2)	20 (11.5)	44 (34.4)	
Receptive	44 (14.6)	33 (19.0)	11 (8.6)	
Both	194 (64.2)	121 (69.5)	73 (57.0)	
Transactional sex in past year				0.25
No	188 (65.5)	114 (68.3)	74 (61.7)	
Yes	99 (34.5)	53 (31.7)	46 (38.3)	
Rectal STIs				0.17
None	238 (78.8)	144 (81.4)	94 (75.2)	
*Neisseria gonorrhoeae*	31 (10.3)	17 (9.6)	14 (11.2)	
*Chlamydia trachomatis*	22 (7.3)	13 (7.3)	9 (7.2)	
Both	11 (3.6)	3 (1.7)	8 (6.4)	
External warts				**< 0.01**
No	222 (72.3)	112 (62.9)	110 (85.3)	
Yes	85 (27.7)	66 (37.1)	19 (14.7)	
No. anal biopsies				**< 0.01**
0	149 (48.5)	62 (34.8)	87 (67.4)	
1	109 (35.5)	76 (42.7)	33 (25.6)	
2	46 (15.0)	38 (21.4)	8 (6.2)	
3	3 (1.0)	2 (1.1)	1 (0.8)	
Anal biopsy results**				**< 0.01**
Benign	127 (41.6)	54 (30.5)	73 (57.0)	
LSIL	157 (51.5)	108 (61.0)	49 (38.3)	
HSIL	21 (6.9)	15 (8.5)	6 (4.7)	

*Abbreviations*: *P* p-value, *No*. number, *STIs* sexually transmitted infections, *LSIL* low-grade squamous intraepithelial lesion, *HSIL* high-grade squamous intraepithelial lesion

*Pearson’s Chi-square and Fisher’s exact tests. Bolded indicates *p* < 0.05

**Worst diagnosis between cytology or histology

**Fig. 1:**
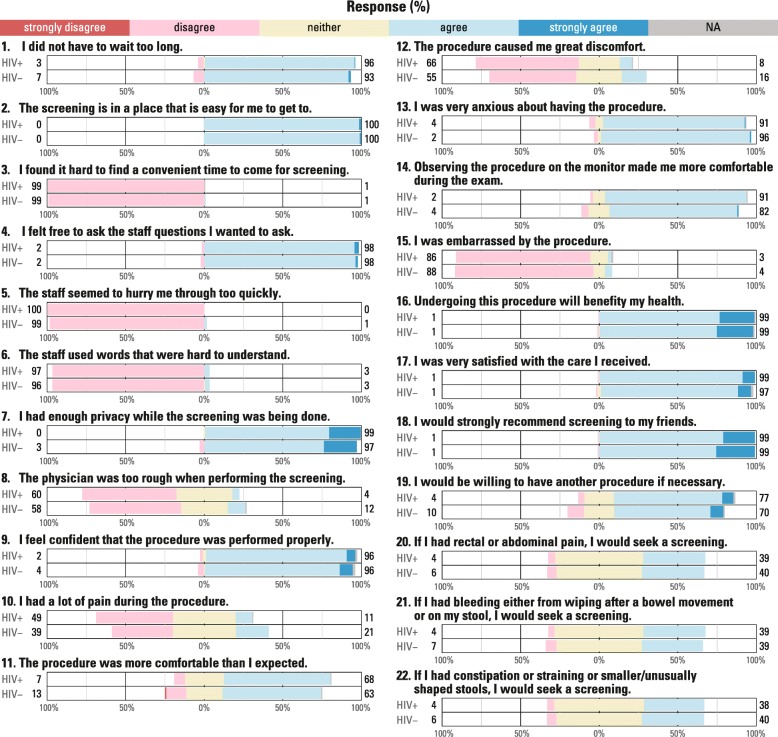
Likert Responses for Each Satisfaction Question Stratified by HIV Infection. Note: Numbers to the right of the bars indicate the total percent with any agreement (agree and strongly agree) and the numbers to the left indicate the total percent with any disagreement (disagree and strongly disagree). The percentages on the x axis indicate the cumulative proportion of Likert responses either in the agree or disagree direction. The neither agree or disagree proportion is centered at 0% and evenly split towards the agree or disagree direction

Participants reported anxiety surrounding the procedure (scaled mean:73), but expressed positive agreement with the convenience/accessibility, staff interpersonal skills, physical surroundings, technical competence, and general satisfaction towards HRA screening (74–76) (Table 2). Willingness to have another procedure if necessary had the lowest reported score within the general satisfaction domain (69). For each domain with multiple questions, concordance was high for the individual responses (range: 0.2 to 6.0%) with the exception of pain/discomfort (12.5%). Responses to each of the questions when averaged within the domains did not differ for those living with or without HIV (Fig. 1) or if they had been recently diagnosed with HIV after adjusting for multiple comparisons (all *p* > 0.006). Overall agreement was also similar irrespective of the number of biopsies (0 to 3) undergone during HRA (all *p* > 0.05) (Table 3). The pain/discomfort and intention to screen if symptomatic had much lower domain scores (scaled means of 57 and 59, respectively) (Table 2). Only 31% of the men agreed HRA did not cause pain and discomfort and less than 40% would seek screening when presented with clinical symptoms of anal cancer (Table 3). Both domains were correlated with lower overall satisfaction (pain and discomfort R_s_ = 0.28, intention to screen if symptomatic R_s_ = 0.63, all *p* < 0.001) Pain/discomfort was also correlated with intention to screen if symptomatic (R_s_ = 0.27, *p* < 0.001).

**Table 2:** Scaled Mean and Standard Deviation for Each Item and 8 Domains within the Satisfaction Survey (*n* = 307)

	Scaled mean^a^	Scaled SD^b^
Expectations		
“I was very anxious about having the procedure”	**72.6**	**7.6**
Convenience and accessibility		
“I did not have to wait too long during my appointment today”	72.6	8.4
“The screening is in a place that is easy for me to get to”	75.2	1.9
*“I [****did not find****] it hard to find a convenient time to come for screening”*^c^	74.8	2.1
Overall	**74.2**	**5.2**
Staff interpersonal skills		
“I felt free to ask the questions I wanted to ask”	74.9	5.1
*“The staff [****did not****] hurry me through too quickly”* ^c^	74.8	2.1
*“The staff [****did not use****] words that were hard to understand”* ^c^	73.6	6.0
Overall	**74.5**	**4.7**
Physical surroundings		
“I had enough privacy while the screening was being done”	79.7	8.2
“Observing the procedure on the monitor made me more comfortable during the exam”	71.2	8.0
Overall	**75.5**	**8.6**
Perceived technical competence		
“I feel confident that the procedure was performed properly”	**75.2**	**7.9**
Pain and discomfort		
*“The physician was [****not****] too rough when performing the screening”* ^c^	49.0	13.4
*“I [****did not have****] a lot of pain during the procedure”* ^c^	49.8	12.8
“The procedure was more comfortable than I expected”	64.3	12.1
*“The procedure [****did not cause****] me great discomfort”* ^c^	50.3	14.2
*“I was [****not****] embarrassed by the procedure”* ^c^	71.2	8.0
Overall	**56.9**	**14.1**
General satisfaction		
“I was very satisfied with the care I received”	76.3	6.4
“Undergoing the procedure will benefit my health”	80.4	8.4
“I would strongly recommend screening to my friends”	80.1	8.8
“I would be willing to have another procedure if necessary”	68.8	12.7
Overall	**76.4**	**10.0**
Intention to screen if symptomatic		
“If I had rectal or abdominal pain, I would seek a screening”	58.7	10.0
“If I had bleeding either from wiping after a bowel movement or on my stool, I would seek a screening”	58.6	10.0
“If I had constipation or straining or smaller/unusually shaped stools, I would seek a screening”	58.6	10.0
Overall	**58.6**	**10.0**

*Abbreviations*: *SD* standard deviation

^a^Scaled mean = (mean-1) × 25 to generate range 0–100

^b^scaled standard deviation = standard deviation*(scaled mean/raw mean)

^c^Reverse coded items are in italics with text in brackets suggesting the reading of the inverse statements

Bolding indicates the overall scaled mean and SD for the 8 evaluated domains

**Table 3:** Distribution of Agreement within 8 Domains by Number of Anal Biopsies Experienced during High Resolution Anoscopy

	Total *N* = 307 *n* (%)	0 *N* = 149 *n* (%)	1 *N* = 109 *n* (%)	2 *N* = 46 n (%)	3 *N* = 3 *n* (%)	*P*^a^
Expectations						0.41
Don’t agree	21 (6.9)	7 (4.7)	10 (9.2)	4 (8.9)	0 (0.0)	
Agree anxious about having the procedure	285 (93.1)	142 (95.3)	99 (90.8)	41 (91.1)	3 (100.0)	
Convenient and acceptable						0.12
Don’t agree	15 (4.9)	9 (6.0)	3 (2.8)	2 (4.4)	1 (33.3)	
Agree did not have to wait long, easy to get to, convenient time	292 (95.1)	140 (94.0)	106 (97.3)	44 (95.6)	2 (66.7)	
Staff interpersonal skills						1.00
Don’t agree	11 (3.6)	6 (4.0)	4 (3.7)	1 (2.2)	0 (0.0)	
Agree could ask questions, not rushed and understood words	296 (96.4)	143 (96.0)	105 (96.3)	45 (97.8)	3 (100.0)	
Physical surroundings						0.91
Don’t agree	9 (2.9)	4 (2.7)	4 (3.7)	1 (2.2)	0 (0.0)	
Agree had enough privacy and observing HRA increased comfort	298 (97.1)	145 (97.3)	105 (96.3)	45 (97.8)	3 (100.0)	
Perceived technical competence						0.51
Don’t agree	11 (3.6)	4 (2.7)	4 (3.7)	3 (6.5)	0 (0.0)	
Agree confident procedure performed properly	296 (96.4)	145 (97.3)	105 (96.3)	43 (93.5)	3 (100.0)	
Pain and Discomfort						0.31
Don’t agree	213 (69.4)	100 (67.1)	74 (67.9)	37 (80.4)	2 (66.7)	
Agree not too rough, not a lot of pain, more comfortable than	94 (30.6)	49 (32.9)	35 (32.1)	9 (19.6)	1 (33.3)	
expected, no great discomfort, not embarrassed						
General Satisfaction						0.72
Don’t agree	5 (1.6)	3 (2.0)	1 (0.9)	1 (2.2)	0 (0.0)	
Agree satisfied, a benefit to my health, strongly recommend to	302 (98.4)	146 (98.0)	108 (99.1)	45 (97.8)	3 (100.0)	
my friends, will have another procedure if needed						
Intention to screen if symptomatic						0.51
Don’t agree	188 (61.2)	87 (58.4)	68 (62.4)	30 (65.2)	3 (100.0)	
Agree will seek screening if have rectal or abdominal pain, bleeding, constipation, straining or unusually shaped stools	119 (38.8)	62 (41.6)	41 (37.6)	16 (34.8)	0 (0.0)	

*Abbreviations*: *P* p-value

^a^Chi-square and Fisher’s exact tests

## Discussion

This cross-sectional study among Nigerian MSM attests to the acceptability of HRA for those who volunteered for their first anal cancer screening even though nearly all were anxious about having the procedure. Our findings also indicate that MSM living with HIV should be prioritized in screening programs, as they had significantly more sexual experience and HPV-associated morbidity relative to participants who were not living with HIV. One of the least agreeable components of the satisfaction survey was the domain related to pain and discomfort which is consistent with other studies [11–13]. Our pain and discomfort domain contained five questions as opposed to two from a similar survey in a previous study of HRA satisfaction at an anal health clinic in the United States, and yet the average score and standard deviation were nearly identical, suggesting the level of discomfort was comparable [11]. Other studies used a 10-point scale and found participants on average reported pain and discomfort levels of 2–4 with 6% of men reporting problematic pain, at a cutoff of ≥7 [6, 12, 13]. When these averages are converted to a 100-point scale, our scaled score of 57 is slightly higher but is not considered problematic (scaled score of 70). More importantly, similar to our findings, there was no correlation between the number of biopsies and pain scores [13]. The pain or discomfort felt during HRA may be from pressure on the sphincter or an exam that took longer than the IANS recommended guidelines of less than 15 min; data not collected in our study [5, 6]. These results emphasize the importance of talking with the patient throughout the process: prior to the procedure, during the exam, and a couple weeks after the exam. Physicians can quantify pain by using a pain scale of 0 to 10 with ≥7 suggestive of problematic pain [6]. Documenting the duration of the HRA exam, particularly for those on the early part of the learning curve, would also help improve the assessment of pain or discomfort and potentially improve its success and sustainability.

But despite these indications of pain and discomfort, our study along with others (80–91%) report a remarkably high proportion of participants who were satisfied with the procedure [12, 13]. However, participants in our study indicated less willingness to undergo future procedures if necessary when compared to previous estimates (92–99%) [12, 13]. These differences may be in part due to our study population being naïve to screening, while other studies included experienced screeners who may have been more likely to respond favorably to future screening as a result of their familiarity with procedures [13]. In another study, only 60% (168/281) of those who were screened completed a willingness questionnaire, potentially inflating the positive responses due to the high proportion of missing data [12]. Regardless of sample differences, open discussions about hesitations with screenings and highlighting the benefits of treating HSIL earlier when it is smaller, if proven to prevent anal cancer, may enhance patient experiences and begin to normalize the importance of anal health among the community.

In order to assess retention of anal cancer-related knowledge after HRA, we added questions about whether HRA would be sought with specific symptoms such as rectal or abdominal pain, bleeding, constipation, and unusually shaped stools. The majority of participants did not agree to seek out screening if faced with these symptoms, especially among those who reported lower levels of general satisfaction. This may be partially attributed to lack of communication regarding anal cancer symptoms from physician to patient during the screening visit or higher levels of pain and discomfort, but there may also be a poor understanding of anal cancer risk in general among MSM. Prior studies of MSM in the United States and Australia have shown that the majority of participants were unaware of anal cancer and its associated risks and qualitatively suggested a perception that HPV-associated cancers only affected women and caused cervical cancer [27, 28]. Knowledge and awareness of HPV as a risk factor for anal cancer may be even more scarce in sub-Saharan Africa where it is rarely reported in cancer registries [29]. A systematic review on HPV-associated cancers from sub-Saharan Africa found 7 of 8 included articles focused on cervical cancer and only 1 included data on anal cancer [30]. The estimates of anal cancer were rare, comprising 1% (21/1627) of the reported male cancers [31]. This highlights the need to increase awareness about the risks and symptoms of anal cancer for healthcare providers and MSM at HIV clinics in sub-Saharan Africa.

This study had a few limitations. First, pain levels and psychological impact of screening procedures were not assessed, and would have enhanced the findings related to pain and discomfort. Questions related to anxiety about cancer were not collected and may have confounded willingness to seek further screening. However, participants overwhelmingly indicated agreed that undergoing screening benefited their health and that they would strongly recommend screening to friends. Because few studies have assessed satisfaction with HRA screening, the questionnaire was minimally modified in order to allow comparison with prior work [11]. The inclusion of general knowledge questions about anal cancer might have provided context to the data collected regarding willingness to seek HRA upon recognition of specific anal cancer symptoms. This study was cross-sectional and could not assess whether pain and discomfort were associated with subsequent screening behavior. The data generated from the responses is only generalizable to those who volunteered to engage in screening and completed the satisfaction survey and does not reflect the views of those who refused screening or those who had not completed the survey. Lastly, even though the questionnaire was self-administered, rather than by a healthcare provider, there may have been positive response bias, which would have inflated measures of satisfaction.

## Conclusions

This study found acceptability with HRA for Nigerian MSM who volunteered to be screened, despite high levels of anxiety. Monitoring pain scales and ensuring the procedure lasts no longer than 15 min could minimize discomfort and would help strengthen the success of a screening program. Integrating anal cancer screening within a community-engaged MSM-friendly clinic offers an opportunity for physicians and healthcare providers to openly discuss clinical symptoms and encourage patients to voice their hesitations about anal cancer screening in order to normalize the concept of men’s anal health in an otherwise highly stigmatizing environment.

## Data Availability

In order to protect the clinic staff and the participants from stigma and criminalization of same sex behavior, the data have not been made publicly available. The data are available from the corresponding author on request.

## Abbreviations

HRA: High resolution anoscopy
MSM: Men who have sex with men
IQR: Interquartile range
CI: Confidence interval
HPV: Human papillomavirus
LSIL: Low-grade squamous intraepithelial lesion
HSIL: High-grade squamous intraepithelial lesion
P: *p*-value
R_s_: Spearman’s rank correlation coefficient
STIs: Sexually transmitted infections
No: Number
